# A tutorial on Bayes Factor Design Analysis using an informed prior

**DOI:** 10.3758/s13428-018-01189-8

**Published:** 2019-02-04

**Authors:** Angelika M. Stefan, Quentin F. Gronau, Felix D. Schönbrodt, Eric-Jan Wagenmakers

**Affiliations:** 1 Department of Psychology, Faculty of Behavioral and Social Sciences, University of Amsterdam, Nieuwe Achtergracht 129-B, 1018WS Amsterdam, The Netherlands; 2 Department of Psychology, Ludwig-Maximilians-Universität München, München, Germany

**Keywords:** Sample size, Design analysis, Bayes factor, Power analysis, Statistical evidence

## Abstract

Well-designed experiments are likely to yield compelling evidence with efficient sample sizes. Bayes Factor Design Analysis (BFDA) is a recently developed methodology that allows researchers to balance the informativeness and efficiency of their experiment (Schönbrodt & Wagenmakers, *Psychonomic Bulletin & Review*, *25*(1), 128–142 2018). With BFDA, researchers can control the rate of misleading evidence but, in addition, they can plan for a target strength of evidence. BFDA can be applied to fixed-N and sequential designs. In this tutorial paper, we provide an introduction to BFDA and analyze how the use of informed prior distributions affects the results of the BFDA. We also present a user-friendly web-based BFDA application that allows researchers to conduct BFDAs with ease. Two practical examples highlight how researchers can use a BFDA to plan for informative and efficient research designs.

## Introduction

A well-designed experiment strikes an appropriate balance between *informativeness* and *efficiency* (Schönbrodt & Wagenmakers, 2018). Informativeness refers to the fact that the ultimate goal of an empirical investigation is to collect evidence, for instance concerning the relative plausibility of competing hypotheses. By carefully planning experiments, researchers can increase the chance of obtaining informative results (Platt, 1964).^1^ Perhaps the simplest way to increase informativeness is to collect more data. Large-N experiments typically result in lower rates of misleading evidence (Ioannidis, 2005), increased stability and precision of parameter estimates (Lakens & Evers, 2014), and a higher replicability of experiments (Etz & Vandekerckhove, 2016). However, sample sizes are subject to the constraints of time, money, and effort^2^—hence, the second desideratum of a well-designed experiment is efficiency: data collection is costly and this drives the need to design experiments such that they yield informative conclusions with as few observations as possible. There is also an ethical argument for efficiency, as it is not just the resources of the experimenter that are at stake, but also the resources of the participants.

In sum, a carefully planned experiment requires that researchers negotiate the trade-off between informativeness and efficiency (Dupont & Plummer, 1990). One useful approach to navigate the trade-off is to conduct a prospective design analysis (Gelman & Carlin, 2013). This method for planning experiments aims to ensure compelling evidence while avoiding sample sizes that are needlessly large. Design analyses can be conducted in both frequentist and Bayesian analysis frameworks (Kruschke, 2013; Cohen, 1992). The most prominent example of prospective design analyses is the frequentist power analysis (Gelman & Carlin, 2014), which builds on the idea of controlling the long-term probability of obtaining significant results given a certain population effect (Cohen, 1988, 1992).

However, the frequentist power analysis has important shortcomings. First, it focuses solely on controlling the rates of false positives and false negatives in significance testing. Other aspects of the informativeness of experiments, such as the expected strength of evidence or the stability and unbiasedness of parameter estimates are neglected (Gelman & Carlin, 2013, 2014; Schönbrodt & Wagenmakers, 2018). Second, frequentist power analysis heavily relies on a priori effect size estimates, which are informed by external knowledge (Gelman & Carlin, 2013). This is problematic insofar as effect size estimates derived from academic literature are likely to be inflated due to publication bias and questionable research practices (Perugini et al., 2014; Simmons et al., 2011; Vevea & Hedges, 1995); to date, there is no optimal method for correcting the biased estimates (Carter et al., 2017). Alternatively, frequentist power analysis can be based on the smallest effect size one wants to detect. However, this approach is used less often in practice (Anderson et al., 2017) and in many cases, defining the minimal important effect size is difficult (Prentice & Miller, 1992).

Recently, several alternative approaches for prospective design analysis have been proposed that can at least partly overcome the shortcomings of frequentist power analysis (e.g., Gelman & Carlin, 2014; Schönbrodt & Wagenmakers, 2018). This paper will focus on one of them: Bayes Factor Design Analysis (BFDA; Schönbrodt & Wagenmakers, 2018), a method based on the concept of Bayesian hypothesis testing and model comparison (Jeffreys, 1961; Kass & Raftery, 1995; Wagenmakers et al., 2010; Wrinch & Jeffreys, 1921).

One of the advantages of BFDA is that it allows researchers to plan for compelling evidence. In a BFDA, the strength of empirical evidence is quantified by Bayes factors (Jeffreys, 1961; Kass & Raftery, 1995). Compared to the conventional power analysis, this means a shift from the focus on the rate of wrong decisions to a broader perspective on informativeness of experiments. Just as a frequentist prospective power analysis, a BFDA is usually conducted in the planning phase of experimental design, that is *before* the data collection starts. However, it could also be conducted and sensibly interpreted “on-the-go” during data collection (Rouder, 2014; Schönbrodt & Wagenmakers, 2018), which can also be considered as an advantage over the current standard approach toward design analyses. Furthermore, BFDA can be applied both to fixed-N designs, where the number of observations is determined in advance, and to sequential designs, where the number of observations depends on an interim assessment of the evidence collected so far (Schönbrodt & Wagenmakers, 2018; Schönbrodt et al.,, 2017).

The present article is directed to applied researchers who are already familiar with the basic concepts of Bayesian data analysis and consider using BFDA for planning experiments.^3^ It has the following objectives: (1) to provide an accessible introduction to BFDA; (2) to introduce informed analysis priors to BFDA which allow researchers more freedom in specifying the expectations about effect size under the alternative hypothesis; (3) to demonstrate how the use of informed analysis priors in BFDA impacts experimental efficiency; (4) to present a user-friendly software solution to conduct BFDAs; and (5) to provide a step-by-step instruction for two common application examples of BFDA. Thus, this tutorial-style paper not only provides an application-oriented introduction to the method proposed by Schönbrodt and Wagenmakers (2018), but also makes new contributions by introducing informed priors and a ready-to-use software solution.

The outline of this paper is as follows. First, we briefly describe Bayes factors and introduce informed analysis priors as a means of incorporating prior information about effect sizes in study designs. Then, we explain the BFDA method in greater detail, addressing both fixed-N and sequential designs. Using two typical analysis priors as an example, we then examine the effects of informed and default analysis priors on the results of a BFDA. Next, we present an interactive web application for BFDA alongside step-by-step instructions for two application examples. The article concludes by discussing possible extensions and implications for empirical research.

## Bayes factors as a measure of strength of evidence

The Bayes factor was originally developed by Harold Jeffreys (1935), building on earlier work published with his co-author Dorothy Wrinch (Wrinch and Jeffreys, 1919, 1921, 1923) as well as on the work of J. B. S. Haldane (Haldane, 1932; Etz & Wagenmakers, 2017). The Bayes factor quantifies the evidence in favor of one statistical model compared to another (Kass & Raftery, 1995). Mathematically, it is defined as the ratio of two marginal likelihoods: The likelihood of the data under the null hypothesis ($\mathcal {H}_{0}$) and the likelihood of the data under the alternative hypothesis ($\mathcal {H}_{1}$).

1 $$ \text{BF}_{10} = \frac{p(\mathbf{D}|\mathcal{H}_{1})}{p(\mathbf{D}|\mathcal{H}_{0})} $$

The Bayes factor can be understood as an updating factor for prior beliefs. For example, when the hypotheses $\mathcal {H}_{0}$ and $\mathcal {H}_{1}$ are deemed equally probable a priori, so that $p(\mathcal {H}_{1}) = p(\mathcal {H}_{0}) = 0.5$, a Bayes factor of BF_10_ = 6 means that after conducting the experiment, $\mathcal {H}_{1}$ is deemed six times more likely than $\mathcal {H}_{0}$ – corresponding to a posterior probability of 86% for $\mathcal {H}_{1}$ and 14% for $\mathcal {H}_{0}$ (Kass & Raftery, 1995).

The Bayes factor plays a central role in Bayesian hypothesis testing (Lewis & Raftery, 1997; Berger, 2006a). Whereas decisions about the rejection of hypotheses are based on *p*-values in frequentist hypothesis testing, decision rules in Bayesian hypothesis testing are based on Bayes factors (Good, 2009, p. 133ff). Usually, defining decision rules implies defining a lower and upper decision boundary on Bayes factors. If a resulting Bayes factor is larger than the upper boundary, it is regarded as good-enough evidence for the alternative hypothesis. If a Bayes factor is smaller than the lower boundary, it is regarded as good-enough evidence for the null hypothesis. If a resulting Bayes factor lies between the boundaries, the evidence is deemed inconclusive (Bernardo & Rueda, 2002). In order to define decision boundaries and interpret evidence from the Bayes factor, researchers often rely on a rough heuristic classification scheme of Bayes factors (Lee & Wagenmakers, 2014). One specification of this classification scheme is depicted in Table 1.

**Table 1:** A heuristic classification scheme for Bayes factors BF_10_ (Lee and Wagenmakers 2013, p. 105; adjusted from Jeffreys, 1961)

Bayes factor	Evidence category
> 100	Extreme evidence for $\mathcal {H}_{1}$
30 - 100	Very strong evidence for $\mathcal {H}_{1}$
10 - 30	Strong evidence for $\mathcal {H}_{1}$
3 - 10	Moderate evidence for $\mathcal {H}_{1}$
1 - 3	Anecdotal evidence for $\mathcal {H}_{1}$
1	No evidence
1/3 - 1	Anecdotal evidence for $\mathcal {H}_{0}$
1/10 - 1/3	Moderate evidence for $\mathcal {H}_{0}$
1/30 - 1/10	Strong evidence for $\mathcal {H}_{0}$
1/100 - 1/30	Very strong evidence for $\mathcal {H}_{0}$
< 1/100	Extreme evidence for $\mathcal {H}_{0}$

A complete Bayesian decision making process also involves the specification of utilities, that is, the value judgments associated with decision options (e.g., Good, 2009; Lindley, 1991; Berger, 1985). However, many Bayesian statisticians focus exclusively on evidence and inference, ignoring the context-dependent specification of utilities. The decision rules for Bayes factors discussed here are decision rules for evidence (i.e., what level of evidence is deemed sufficiently compelling?). These rules may be influenced by prior model plausibility and by utilities, but these elements of the decision process are not specified separately and explicitly.

The use of Bayes factors as a measure of evidential strength provides several advantages. First, Bayes factors can quantify evidence for $\mathcal {H}_{0}$ and $\mathcal {H}_{1}$ (Kass & Raftery, 1995). This means that in contrast to *p*-values, they can distinguish between absence of evidence and the evidence of absence (Altman & Bland, 1995; Dienes, 2014). Bayes factors also do not require the two models to be nested, which increases researchers’ freedom in formulating relevant hypotheses (Kass & Raftery, 1995). Another advantage of Bayes factors is that their interpretation remains meaningful despite optional stopping (Rouder, 2014). This allows sequential research designs where the decision about the continuation of sampling depends on the value of the Bayes factor (Schönbrodt et al., 2017).

## Bayes factors with informed priors

As stated before, the Bayes factor is defined as the ratio of the marginal likelihood of the data under the null and the alternative hypothesis, respectively. In the simplest case both hypotheses, $\mathcal {H}_{0}$ and $\mathcal {H}_{1}$, are point hypotheses which means that they assume that the parameter in question (e.g., the effect size) takes one specific value (e.g., “$\mathcal {H}_{0}$: The parameter *𝜃* is 0.”, “$\mathcal {H}_{1}$: The parameter *𝜃* is 1”). In this case, the Bayes factor is equivalent to a simple likelihood ratio. However, hypotheses often rather assume that the parameter in question lies within a certain range of values (e.g., $\mathcal {H}_{1}$: *“The parameter𝜃*_*k*_*is larger than 0, and smaller values of𝜃*_*k*_*are more likely than larger values.”*). In this case, the hypothesis is specified as a distribution that assigns a probability (density) to parameter values. We call this distribution the prior distribution on parameters. The marginal likelihood can then be calculated by integrating over the parameter space, so that

2 $$ p(\mathbf{D}|H_{k}) = \int{p(\mathbf{D}|\theta_{k}, H_{k})\pi(\theta_{k}|H_{k}) \text{d}\theta_{k}}, $$

where *𝜃*_*k*_ is the parameter under *H*_*k*_, *π*(*𝜃*_*k*_|*H*_*k*_) is its prior density, and *p*(**D**|*𝜃*_*k*_,*H*_*k*_) is the probability density of the data **D** given a certain value of the parameter *𝜃*_*k*_ (Kass & Raftery, 1995; Rouder et al., 2009).

Opinions differ as to how much information should be included in the prior distribution on parameters, that is, *π*(*𝜃*_*k*_|*H*_*k*_). So-called “objective” Bayesians favor non-informative distributions which do not put too much weight on single parameter values and are constructed to fulfill general desiderata (Rouder et al., 2009; Ly et al., 2016). Objective Bayesians advocate default prior distributions that do not rely on the idiosyncratic understanding of a theory and on the potentially flawed subjective elicitation of an informative prior distribution (Berger, 2006b). In contrast, so-called “subjective” Bayesians argue that “the search for the objective prior is like the search for the Holy Grail” (Fienberg, 2006, p. 431). Subjective Bayesians claim that no statistical analysis can be truly objective, and they critique objective Bayesians for using prior distributions that are at best inaccurate reflections of the underlying theory (Goldstein, 2006).

**Fig. 1:**
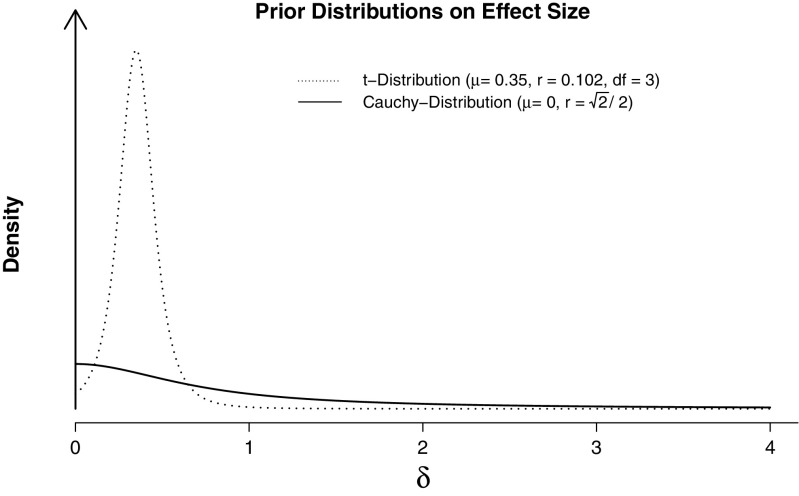
Informed and default prior distribution on effect size *δ* used in this article. Default prior distribution proposed by Rouder et al., (2009) for Bayesian *t*-tests, informed prior distribution elicited by Gronau et al., (2017) for a replication study in social psychology. Figure available under a CC-BY4.0 license at https://osf.io/3f5qd/

In its original version, BFDA was applied to Bayesian hypothesis testing with objective priors (Schönbrodt & Wagenmakers, 2018). In this paper, we introduce subjective priors to BFDA and investigate how their use impacts design efficiency. As in the original paper, we will use Bayesian *t*-tests with directional hypotheses to illustrate the procedure. As proposed by Rouder et al., (2009), we will use a central Cauchy distribution with a scale parameter of $r = \sqrt {2}/2$ as a default (“objective”) prior distribution on effect size *δ*. This prior is also a default setting in current statistical software covering Bayesian statistics like the *BayesFactor* package (Morey and Rouder, 2015) for the R Environment for Statistical Computing (R Development Core Team, 2011) and JASP (The JASP Team, 2018). The informed prior distribution investigated in this paper was originally elicited by Gronau et al., (2017) for a replication study in the field of social psychology and, in our opinion, can serve as an example for a typical informed prior for the field of psychology. It is a shifted and scaled t-distribution with a location parameter of *μ* = 0.35, 3 degrees of freedom, and a scale parameter of *r* = 0.102. Both prior distributions (objective and informed) are depicted in Fig. 1.

## Bayes Factor Design Analysis for fixed-N and sequential designs

One important step of experimental planning is to determine the sample size of the experiment. In fixed-N designs, the sample size is determined before conducting an experiment based on pre-defined desiderata for the expected strength of evidence and the probability of decision errors (Schönbrodt and Wagenmakers, 2018). In sequential designs, instead of a fixed sample size a decision rule is set before the start of the experiment. This decision rule determines when the sampling process will be stopped. Researchers can decide at every stage of the experiment on the basis of the decision rule whether to (1) accept the hypothesis being tested; (2) reject the hypothesis being tested; or (3) continue the experiment by making additional observations (Wald, 1945). For example, a researcher might aim for a strength of evidence of 6, and thus collect data until the Bayes factor (BF_10_) is larger than 6 or smaller than 1/6. Sequential designs are particularly easy to use in a Bayesian framework since the Bayes factor is robust against optional stopping, so no correction mechanism needs to be employed for looking at the data before the experiment is concluded (Rouder, 2014; Schönbrodt et al., 2017).^4^ Additionally, it is guaranteed that finite decision boundaries will eventually be reached, since the Bayes factor approaches 0 or *∞* when the data are overwhelmingly informative which happens when the sample size becomes very large (a property called *consistency*; Ly et al.,, 2016).

BFDA can help researchers plan experiments with both fixed-N and sequential designs. The target outcomes of BFDAs depend on the choice of design. In fixed-N designs, a BFDA provides researchers with a distribution of Bayes factors, that is, of the expected strength of evidence. Large Bayes factors pointing towards the wrong hypothesis can be interpreted as misleading evidence because they likely lead to decision errors. Researchers define “large Bayes factors” based on two boundaries, e.g., “all Bayes factors that are smaller than 1/10 or larger than 10 are counted as strong evidence for the null and alternative hypothesis, respectively”.

For sequential designs, the BFDA results in a large number of sampling trajectories. Each sampling trajectory mimics one possible experimental sequence, for example “a researcher starts with ten participants and adds one participant at a time until the Bayes factor of the collected data is larger than 6, which happens at the 21st participant”. In sequential designs, the end point of the sampling trajectory, which is the final sample size of the experiment, is a random variable. Hence, the most interesting information a BFDA can provide in sequential designs is a probability distribution of this random variable, that is a probability distribution of final sample sizes. Additionally, a BFDA can estimate the percentage of trajectories that will arrive at the “wrong” boundary, that is at the upper boundary when the null hypothesis is true or at the lower boundary when the alternative hypothesis is true. This percentage of trajectories can be interpreted as rate of misleading evidence in sequential designs (Schönbrodt & Wagenmakers, 2018).

**Fig. 2:**
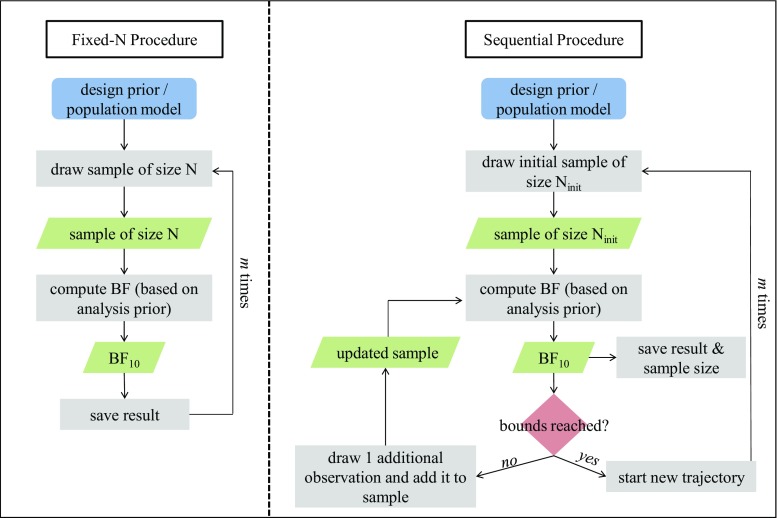
Flowchart of the BFDA simulation process. *Rectangles* show actions, *diamonds* represent decisions, and *parallelograms* depict outputs. Typically, the simulation is conducted once under the null and once under the alternative hypothesis. Figure available under a CC-BY4.0 license at https://osf.io/3f5qd/

BFDA is based on Monte Carlo simulations. The simulation procedure is displayed in a flowchart in Fig. 2 and can be summarized as follows Schönbrodt and Wagenmakers (2018):

1. Simulate a population that reflects the effect size under $\mathcal {H}_{1}$. If the effect size under $\mathcal {H}_{1}$ is composite (e.g., $\mathcal {H}_{1}: \delta \sim t(0.35, 3, 0.102)$), draw a value of *δ* from the respective distribution. In the example analysis used in this article, we simulate two subpopulations with normal distributions. In the following sections, we will refer to simulated populations with effect sizes of *δ* = 0.2, *δ* = 0.35, *δ* = 0.8, and *δ* = 0.
2. Draw a random sample of size N from the simulated subpopulations. For the fixed-N design, the sample size corresponds to the pre-determined sample size. For the sequential design, the initial sample size corresponds to a minimum sample size, which is either required by the test (e.g., for an independent-sample *t*-test, this sample size is equal to 2 observations per group) or set to a reasonable small number. In our example, we chose a minimum sample size of ten observations per group.
3. Compute the Bayes factor for the simulated data set. In sequential design, increase the sample size by 1 if the Bayes factor does not exceed one of the decision thresholds and compute the resulting Bayes factor with the new sample. Continue doing so until either of the thresholds is reached (e.g., BF_10_ < 1/6 or BF_10_ > 6).
4. Repeat steps 1 to 3 *m* times, e.g., *m* = 10,000.
5. In order to obtain information on the design under the $\mathcal {H}_{0}$, steps 1 to 4 must be repeated under $\mathcal {H}_{0}$, that is, with two populations that have a standardized mean difference of *δ* = 0.

For the fixed-N design, the simulation results in a distribution of Bayes factors under $\mathcal {H}_{1}$ and another distribution of Bayes factors under $\mathcal {H}_{0}$. To derive rates for false-positive and false-negative evidence, one can set decision thresholds and retrieve the probability that a study ends up in the “wrong” evidential categories according to these thresholds. For the sequential design, the simulation results in a distribution of N that is conditional on the set evidence thresholds. The rates of misleading evidence can be derived by analyzing the percentage of cases which fell into the “wrong” evidential category, that is, arrived at the wrong boundary.

## Bayes Factor Design Analysis with informed priors

As in earlier work (O’Hagan et al., 2005; Walley et al., 2015), Schönbrodt and Wagenmakers (2018) distinguish “design priors” and “analysis priors”. Both are prior distributions on parameters, but have different purposes. Design priors are used before data collection as data generating model to simulate (sub)populations. Analysis priors are used for Bayesian statistical analysis of the collected data (Schönbrodt & Wagenmakers, 2018; O’Hagan et al., 2005).

As both kinds of priors represent beliefs about the true state of nature under the hypotheses in question, some researchers may feel this distinction is artificial. This holds especially true when design priors are distributions, that is, when simulated effect sizes are generated from distributions. The introduction of informed priors to BFDA makes the distinction unnecessary and can therefore yield more intuitive results.

The following sections explore the impact of the choice of priors on design efficiency and informativeness in greater depth. It is important to note that in practice the choice of priors should always be based on theoretical considerations and not only on their influence on design properties. However, we will show that the choice of priors is an important aspect of a design that needs to be considered in the planning of experiments.

## Bayes Factor Design Analysis for fixed-N designs

In the fixed-N design, sample size and expected population effect size are defined by the researcher. Questions that can be answered by a BFDA for this design are:

- What Bayes factors can I expect?
- What is the probability of misleading evidence?
- What sample size do I need to obtain true positive or true negative evidence with a high probability?

In the following sections, we will tackle these questions and explore the effects of the choice of analysis prior for different design priors and sample sizes. In our examples, we use four different effect sizes as a design prior: *δ* = 0 as data generating model under $\mathcal {H}_{0}$, *δ* = 0.2 as a small effect size which is somewhat larger than what would be expected if there was a null effect but somewhat smaller than what would be expected from the informed analysis prior distribution, *δ* = 0.35 as a true effect size which perfectly matches the mode of the informed analysis prior distribution, and *δ* = 0.8 as a large effect size which is still within the typical range for the field of psychology (Perugini et al., 2014), but which is considerably larger than what would be expected from the informed analysis prior. Additionally, we will consider sample sizes between *N* = 10 and *N* = 500 observations per group, which is typical for the field of psychology (Fraley & Vazire, 2014; Marszalek et al., 2011).

### Expected Bayes factors

**Fig. 3:**
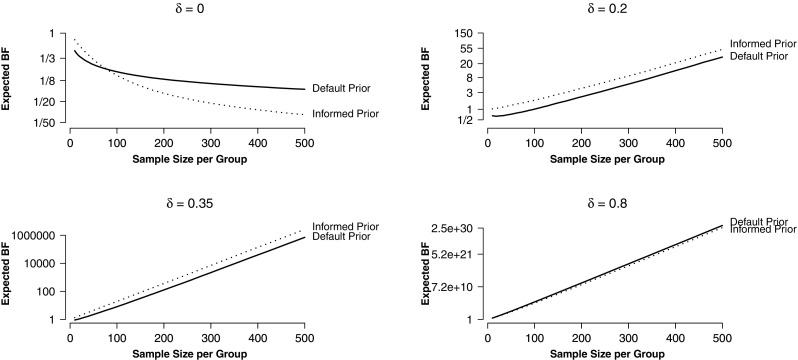
Expected Bayes factors for different true effect sizes. Expected Bayes factors are defined as the raw Bayes factors corresponding to the mean log Bayes factors for a specific sample size. Evidence accumulates more slowly when the null hypothesis is true (*δ* = 0) than when it is false. Figure available under a CC-BY4.0 license at https://osf.io/3f5qd/

As can be seen in Fig. 3, expected Bayes factors increase with increasing sample size if the true effect size is larger than zero. If there is no difference between groups (*δ* = 0), the expected Bayes factor approaches zero. This implies that the mean log Bayes factor decreases to −*∞* when sample size increases. In other words, when sample size increases, so does the evidence for the true data generating model. However, evidence for the null hypothesis accumulates at a slower rate than evidence for the alternative hypothesis (Johnson & Rossell, 2010). In Fig. 3, this can be seen from the smaller gradient of the panel for *δ* = 0.

As expected, the choice of the analysis prior influences the expected Bayes factor. If the true effect size lies within the highest density region of the informed prior distribution (e.g., *δ* = 0.2,*δ* = 0.35), the evidence accumulates faster when the informed prior distribution is used compared to when a default prior is used. In contrast, if the true effect size is much larger than the mode of the informed prior distribution (e.g., *δ* = 0.8), the expected Bayes factor for a given sample size is slightly larger for the default prior approach. This can be understood as a higher “riskiness” of the choice of the informed prior. Researchers who plan a study can be more conservative by choosing broader analysis priors—these are less efficient in general (e.g., they yield lower Bayes factors for the same sample size) but more efficient when the true effect size does not match the prior expectations. Alternatively, researchers who already have specific expectations about the population parameter can make riskier predictions by choosing informed prior distributions—these are potentially more efficient, but only when the true effect size matches the expectations.

When data are generated under the null hypothesis (top left panel in Fig. 3), there is no unconditional efficiency gain for informed or default analysis priors. If the sample size is smaller than 100 observations per group, the expected evidence for the null hypothesis is stronger in the default prior approach. For larger sample sizes, the informed prior approach yields stronger evidence.

### Probability of misleading evidence

Rates of misleading evidence can only be determined in a decision-making context. These rates are dependent on the choice of cut-off values that guide the decision towards $\mathcal {H}_{0}$ or $\mathcal {H}_{1}$. In a Bayesian framework, cut-off values are usually determined in terms of Bayes factors by choosing an upper and a lower decision boundary. Typically, these boundaries are chosen symmetrically. This means that the upper and lower boundary are defined as *b*_*BF*_ and 1/*b*_*BF*_, respectively.

**Fig. 4:**
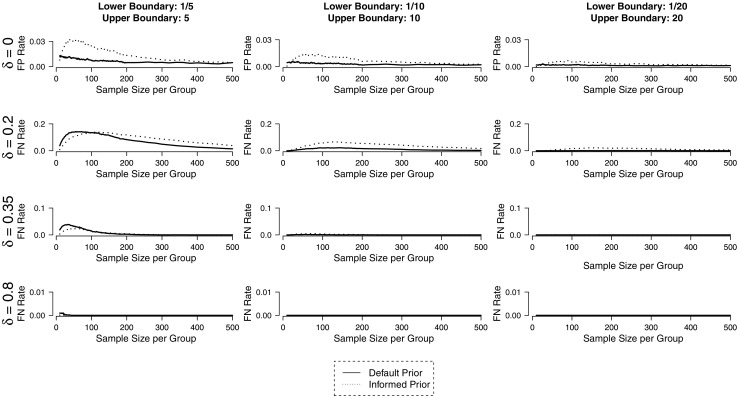
Rates of false-positive (FP) and false-negative (FN) evidence in fixed-N design for different true effect sizes. If informed analysis priors are used, less FN evidence occurs. If default analysis priors are used, less FP evidence occurs. Figure available under a CC-BY4.0 license at https://osf.io/3f5qd/

Figure 4 shows the expected rates of misleading evidence for symmetric boundaries given different true effect sizes. What may come as a surprise is that the rate of misleading evidence does not decrease continuously with increasing sample size. This happens because evidence is mostly inconclusive for small sample sizes, that is, the Bayes factor is larger than the lower boundary but smaller than the upper boundary. For example, if *δ* = 0 and we choose N = 10 per group and decision boundaries of $\frac {1}{10}$ and 10, the resulting evidence is inconclusive in over 99% of the cases. Therefore, the evidence is misleading in only a very small number of cases, but it also does not often motivate any decision either. This illustrates an important difference compared to standard frequentist statistics: While there are mostly only two possible outcomes of an experiment in frequentist statistics, namely, a decision for or against the null hypothesis, the absence of evidence is a possible third outcome in Bayesian hypothesis testing.^5^

Analogous to frequentist statistics, rates of misleading evidence decrease as effect size and sample size increase. In addition, the choice of decision boundaries also influences the quality of decisions: the higher the boundaries, the lower the rates of misleading evidence. Figure 4 shows that the informed and default prior approach have distinct properties in terms of error rates. While rates of false-positive evidence are mostly lower for the default analysis prior, rates of false-negative evidence are mostly lower for the informed analysis prior. This may be important when planning a study because sample size or decision boundaries may need to be adjusted accordingly depending on the prior distribution.

### Sample sizes to obtain true positive or true negative evidence with a high probability

An important characteristic of a good experiment is its ability to provide compelling evidence for a hypothesis when this hypothesis is true. In fixed-N designs this can be achieved by determining the number of observations needed to obtain strong positive evidence (when $\mathcal {H}_{1}$ is true) or strong negative evidence (when $\mathcal {H}_{0}$ is true) with a high probability. Just as rates of misleading evidence, these rates of true positive and true negative evidence also depend on the chosen Bayes factor boundaries. The question here is: “Which sample size is required to obtain a Bayes factor that exceeds the ‘correct’ boundary with a high probability, say 80%?”

**Fig. 5:**
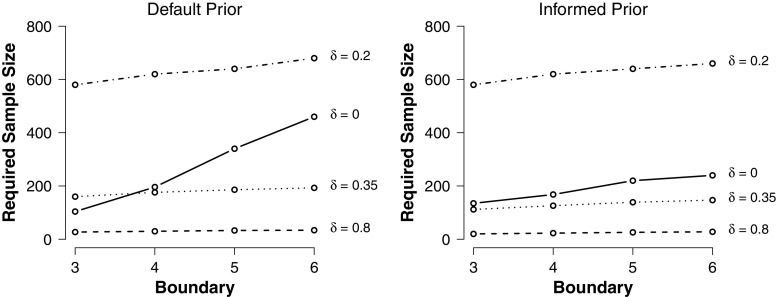
Required sample sizes per group to obtain true positive (if $\mathcal {H}_{1}$ is true) or true negative (if $\mathcal {H}_{0}$ is true) evidence with an 80% probability for symmetric decision boundaries between 3 and 6 and different effect sizes *δ*. Largest sample sizes are required if the true effect size is small but non-zero. Figure available under a CC-BY4.0 license at https://osf.io/3f5qd/

If the design prior is larger than zero, this critical sample size can be obtained by repeatedly conducting a fixed-N BFDA for increasing sample sizes and computing the 20% quantile of each Bayes factor distribution. The critical sample size is reached when the 20% quantile of the Bayes factor distribution exceeds the Bayes factor boundary (this means that for this sample size 80% of the Bayes factors are larger than the boundary). Figure 5 depicts the required sample sizes for symmetric boundaries between 3 and 6 for different true effect sizes when using either a default or an informed analysis prior.

Clearly, if the true effect size is large, smaller sample sizes are sufficient to detect an effect. For example, when the true effect size is *δ* = 0.35 and the default analysis prior is used, 185 observations per group are needed to obtain a Bayes factor larger than 5 with a probability of 80%. In contrast, only 33 observations per group are needed to obtain the same evidence strength with the same probability for *δ* = 0.8. When the null hypothesis is true in the population (*δ* = 0), 340 observations are needed to gain the same strength of evidence in favor of the null. The largest sample sizes are required when the true effect size lies close to zero but does not equal zero. The reason is that it is difficult to determine whether in this region $\mathcal {H}_{0}$ or $\mathcal {H}_{1}$ was the data generating process, so Bayes factors will often meander between the boundaries or arrive at the wrong boundary. There are also perceivable differences between the default and informed prior approach. In general, smaller samples are required if an informed analysis prior is used. This corroborates the findings mentioned in earlier sections of this paper, that the informed prior approach is more diagnostic for smaller sample sizes.

In practice, if researchers want to plan for strong evidence independently of whether the null or the alternative hypothesis is valid in the population, they can compute the critical sample size for both hypotheses and plan for the larger sample size. For instance, if 185 observations per group are needed to obtain true positive evidence in 80% of the time (if H1 is true) and 340 observations per group to obtain true negative evidence in 80% of the time (if H0 is true), it is sensible to aim for the higher sample size because before the experiment is conducted it is not clear whether the effect size is zero or non-zero in the population. Of course, researchers can also set different criteria for decision bounds or true evidence rates depending on the hypothesis.

## Bayes Factor Design Analysis for sequential designs

**Fig. 6:**
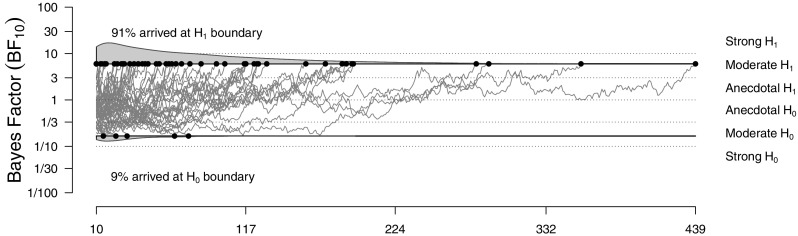
An example for the sequential sampling procedure (true effect size: *δ* = 0.35, symmetric boundaries: {$\frac {1}{6}, 6$}, analysis prior: default). Misleading evidence occurs mainly when trajectories end early. Figure available under a CC-BY4.0 license at https://osf.io/3f5qd/

In sequential designs, sampling is continued until the desired strength of evidence is reached; consequently, the evidence is now fixed. However, prior to conducting the experiment, it is unknown at which sample size these boundaries will be reached and how often Bayes factor trajectories arrive at the wrong boundary (see Fig. 6). Thus, we can ask the following two questions in a BFDA for sequential designs:

1. Which sample sizes can be expected?
2. What is the probability of misleading evidence?

### Expected sample sizes

**Fig. 7:**
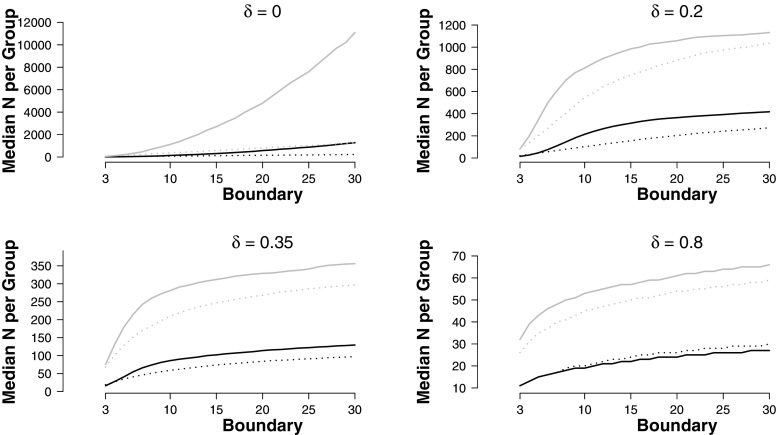
Median sample sizes per group and 95% quantiles of the sample size distribution in sequential design for different symmetric decision boundaries for Bayes factors with informed and default analysis priors. *Black lines* are for medians, *grey lines* for 95% quantiles. *Solid lines* represent the default prior, *dotted lines* the informed prior. Figure available under a CC-BY4.0 license at https://osf.io/3f5qd/

Since the final sample size in a sequential design is a random variable, one of the most urgent questions researchers have when planning sequential designs is what sample size they can expect. BFDA answers this question with a distribution of sample sizes. The quantiles of this distribution can be used to plan experiments. For example, researchers might be interested in a plausible estimate for the expected sample size. Since the distribution is skewed, the median provides a good measure for this central tendency. When planning for resources, researchers might also be interested in a plausible estimate for the maximum sample size they can expect. In this case, it is reasonable to look at a large quantile of the distribution, for example the 95% quantile. Figure 7 displays the median and the 95% quantile of sample size distributions for symmetric decision boundaries between 3 and 30 (corresponding to lower boundaries from $\frac {1}{3}$ to $\frac {1}{30}$ and upper boundaries from 3 to 30). For small to medium effect sizes, the required sample sizes are clearly smaller when informed analysis priors are used. For large effect sizes (e.g., *δ* = 0.8), the default analysis prior approach is more efficient. However, for large effect sizes the required sample sizes are small in general, and consequently the efficiency gain is relatively modest. When the null hypothesis is true in the population, there is a striking difference in the 95% quantile of the sample size distribution. This shows that in this case it is more likely that it takes very long until the Bayes factor trajectory reaches a threshold when the default analysis prior is used.

### Probability of misleading evidence

In sequential designs, misleading evidence is defined as Bayes factor trajectories that arrive at the “wrong” decision boundary, that is, at the $\mathcal {H}_{0}$ boundary when $\mathcal {H}_{1}$ is correct and vice versa (Schönbrodt et al., 2017). As can be seen in Fig. 6, misleading evidence occurs mainly when Bayes factor trajectories end early, that is when sample sizes are still small.^6^

**Fig. 8:**
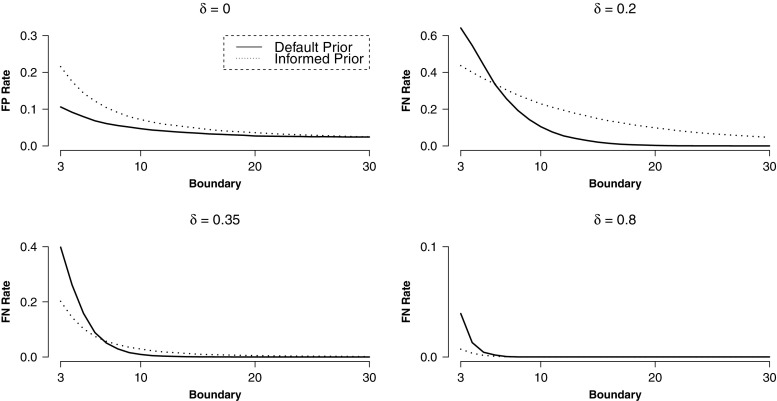
Rates of misleading evidence in sequential design for different decision boundaries and true effect sizes. Figure available under a CC-BY4.0 license at https://osf.io/3f5qd/

Figure 8 displays rates of misleading evidence in sequential designs. One can observe a rapid decline in error rates when symmetrical decision boundaries are raised from 3 to about 10. When they are further increased, error rates improve only marginally. This finding is important for balancing informativeness and efficiency in the planning stage of an experiment with sequential designs. To ensure informativeness of experiments, rates of misleading evidence can be controlled, but this usually comes at the cost of efficiency in terms of sample size. In sequential designs, a good balance can be found by increasing decision boundaries (and thereby sample sizes) until error rates change only marginally.

When comparing designs with default and informed analysis priors, the same pattern as in the fixed-N design can be observed. While models with informed priors yield comparatively less false-negative evidence, models with default priors yield less false-positive evidence. However, these differences disappear with large decision boundaries.

## A Shiny App for Bayes Factor Design Analysis

In the previous sections, we have highlighted how, in the planning stage of an experiment, a BFDA can help balance important aspects of informativeness and efficiency, namely the expected strength of evidence, the rates of misleading evidence, and the (expected) sample size. Yet, conducting a BFDA may be troublesome to some researchers because (a) it requires advanced knowledge of programming as it is not yet an integral part of statistical software; and (b) Monte Carlo simulations are computationally intensive and therefore time-consuming. In the second part of this article, we therefore want to introduce a user-friendly app which makes BFDA accessible to researchers without programming experience or access to high-performance computers. First, we provide a short overview on the app, then we demonstrate its application in two examples by giving step-by-step instructions on how to use the app to answer two questions on design planning.

We used the *Shiny* package for R to create the BFDA app (Chang et al., 2017). Shiny is an open source R package that provides a web framework for building dynamic web applications with an R-based graphical user interface. The core of the app is a large database of precomputed Monte Carlo simulation results, which allows users to conduct a BFDA quickly. Depending on the available computer power, one simulation can easily take an entire day, and our database solution overcomes this computational hurdle. In total, we conducted 42 different simulations spanning 21 different true effect sizes. Our simulation code is based on the *BFDA* package for R (Schönbrodt, 2016) and is available under CC-BY4.0 license on https://osf.io/3f5qd/.

The app consists of two parts, one for fixed-N designs and the other for sequential designs. The app allows users to conduct all analyses mentioned in the previous sections; in addition, it provides summary plots for a preliminary analysis as well as additional figures for single-case scenarios (e.g., distribution of Bayes factors in fixed-N design for a specific sample size). Moreover, it allows users to download dynamic, time-stamped BFDA reports.

Users can choose effect sizes between *δ* = 0.2 and *δ* = 1.2 under the alternative hypothesis, symmetric decision boundaries between 3 and 30, and (for the fixed-N design) sample sizes between 10 and 200 per group. This parameter range is typical for the field of psychology (Fraley & Vazire, 2014; Lee & Wagenmakers, 2014; Marszalek et al., 2011; Perugini et al., 2014). Users can also evaluate how their experimental design behaves when the null hypothesis is true, that is, when *δ* = 0. They can also choose between the two analysis priors that are used throughout this article (see Fig. 1).

The BFDA app is an interactive and open-source application. This means that users can decide what information should be displayed and integrated in the analysis report. The source code of the app as well as all simulated results are openly accessible and can be downloaded from GitHub (https://github.com/astefan1/Bayes-Factor-Design-Analysis-App) and the OSF platform (https://osf.io/3f5qd/), respectively. This allows users who want to adapt the BFDA simulation when it does not meet their needs.

In the following sections, we introduce two application examples for the BFDA app, tackling typical questions that a researcher could have in the planning stage of an experiment.

## Two step-by-step application examples

### Fixed-N design: can I find evidence for the null?

One of the main advantages of the Bayesian method is that it makes it possible to quantify evidence in favor of the null hypothesis (Altman & Bland, 1995; Dienes, 2014). Yet, finding evidence for the null hypothesis is typically more difficult than for the alternative hypothesis (Jeffreys, 1961, p. 257). This is also illustrated in Fig. 3, which shows that as sample size increases, Bayes factors decrease at a lower rate for *δ* = 0 than they increase when *δ* > 0. This implies that larger sample sizes are needed to gain the same strength of evidence for $\mathcal {H}_{0}$ as for $\mathcal {H}_{1}$.

This leads to a potential asymmetry in evidential value of experiments: If the sample size of an experiment is small, it may be likely to gain strong evidence for the alternative hypothesis if $\mathcal {H}_{1}$ is true but highly unlikely to gain strong evidence for the null hypothesis if $\mathcal {H}_{0}$ is true. Thus, for small sample sizes the possible informativeness of the Bayesian method is not fully exploited, because it is only possible to distinguish between “evidence for $\mathcal {H}_{1}$” and “inconclusive evidence”.

In this example, we use BFDA to assess whether it is possible to gain strong evidence for the null hypothesis in a particular research design. We consider the following scenario: A recent study on researchers’ intuition about power in psychological research found that roughly 20% of researchers follow a rule-of-thumb when designing experiments (Bakker et al., 2016). The authors specifically mention the “20 subjects per condition” rule, which states that 20 observations per cell guarantee sufficient statistical power (Simmons et al., 2011). In an independent sample *t*-test this corresponds to two groups of 20 observations each.

**Fig. 9:**
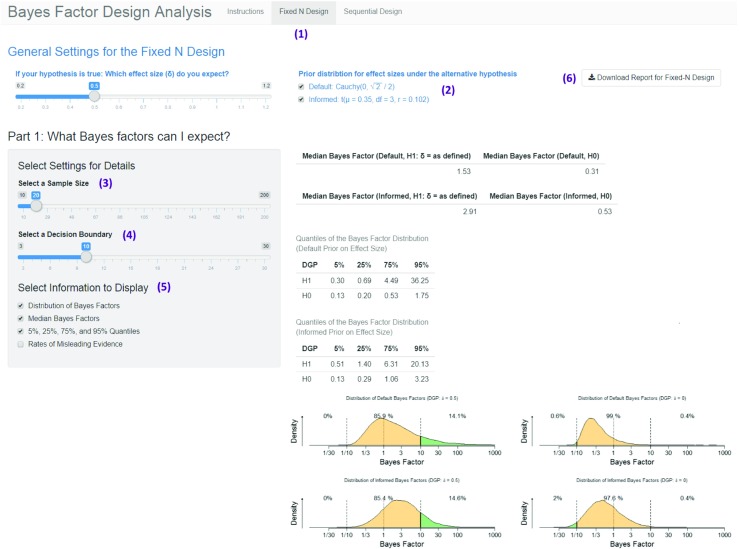
Screenshot from the Bayes Factor Design Analysis (BFDA) app. *Purple numbers* are added to describe the procedure of answering the question: Is it possible to find (strong) evidence for the null with a specific sample size?

Is a sample size of 20 observations per group sufficient to obtain strong evidence for the null? We will answer this question step-by-step by using the BFDA app (see Fig. 9). The app can be accessed under http://shinyapps.org/apps/BFDA/.

1. Choosing a design: As our question involves a specific sample size, we need to choose the tab for fixed-N design.
2. Choosing the priors: In our example, we did not specify whether we want to use default or informed analysis priors. However, it could be interesting to compare whether the results are robust to the choice of prior, so we will select both in this example. The selection of the design prior (expected effect size under the alternative hypothesis, see slider on the left) is not relevant in our example, because we are solely interested in the null hypothesis, that is *δ* = 0.
3. Choosing a sample size: As defined in the question, we are interested in a design with 20 observations per group, so the slider should be adjusted to 20.
4. Choosing a decision boundary: We will choose a boundary of 10, which demarcates the threshold between moderate and strong evidence according to the classification by Lee and Wagenmakers (2014, see Table 1). This choice of boundaries corresponds to an upper boundary of 10 and a lower boundary of $\frac {1}{10}$.
5. Select information that should be displayed: We are interested in the expected distribution of Bayes factors. Thus, we will select the options “Distribution of Bayes Factors” (yielding graphic output), “Median Bayes Factors” (as an estimate for the expected Bayes factor), and “5%, 25%, 75%, and 95% Quantiles” (to get a numeric summary of the entire distribution)
  The results of the precomputed Monte Carlo simulations are displayed in the panel on the right of Fig. 9. On top, a table with the medians of the Bayes factor distribution is displayed. For the informed analysis prior, the median Bayes factor under $\mathcal {H}_{0}$ is 0.53, and for the default analysis prior it is 0.31. The table underneath shows the 5%, 25%, 75%, and 95% quantiles of the Bayes factor distribution. We can see that for both analysis priors, the 5% quantile equals 0.13. The figures at the bottom show that in most cases, the evidence is inconclusive given the selected boundaries as indicated by the large yellow areas. Bayes factors smaller than $\frac {1}{10}$ can only be expected in 0.6% of the cases for default priors and in 2% of the cases for informed priors. Combining these results, one can conclude that it is highly improbable that a Bayesian *t*-test with *N* = 20 per group yields strong evidence for the null hypothesis, even if the null hypothesis is true. The sample size is too small to fully exploit the advantages of the Bayesian method and should therefore be increased.
6. Download report: To store the results, a time-stamped report in pdf format can be downloaded by clicking on the download button on the right top of the page. The report contains the results as well as the selected options for the design analysis. The report for our first application example can be downloaded from https://osf.io/3f5qd/.

### Sequential design: how large will my sample be?

In sequential designs, sampling continues until a certain decision boundary is reached. Thus, researchers cannot know the exact sample size prior to the study. However, planning for financial and organizational resources often requires at least a rough idea about the final sample size. In our second example we therefore want to show how a BFDA can answer the question: “How large will my sample be?”

**Fig. 10:**
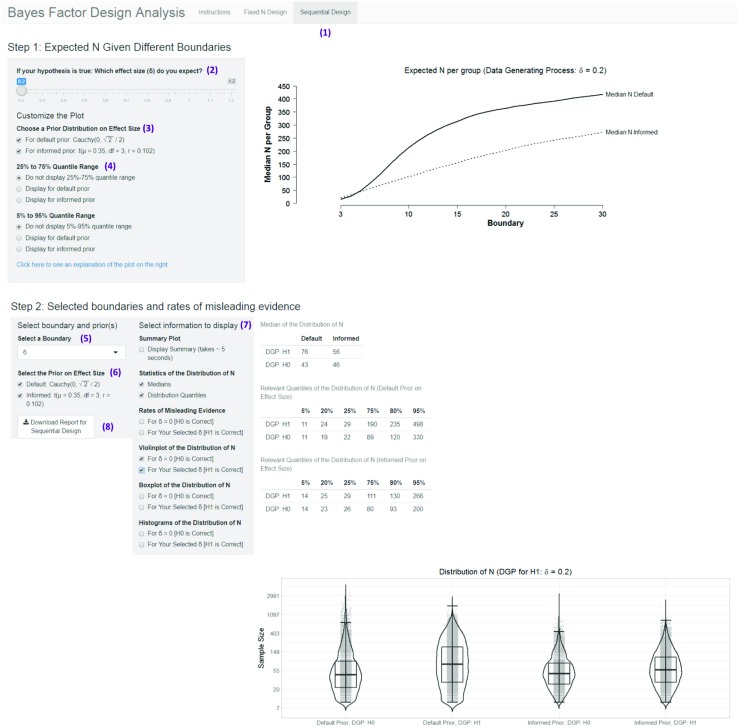
Screenshot from the Bayes factor design analysis (BFDA) app. *Purple numbers* are added to describe the procedure of answering the question: “How large will my sample size be in a sequential design given certain decision boundaries and true effect sizes?” Figure available under a CC-BY4.0 license at https://osf.io/3f5qd/

We will again explain how to use the BFDA app to answer the question step-by-step (see Fig. 10). The app can be accessed under http://shinyapps.org/apps/BFDA/.

1. Choosing a design: As we are interested in the expected sample size in a sequential design, we need to choose the sequential design tab.
2. Choosing a design prior: Try to answer the question: “What effect size would you expect if your alternative hypothesis is true?” This is the same question that you have to ask yourself if you want to construct a reasonable informed analysis prior. So one possibility to choose the effect size is to choose the mode of the informed analysis prior. However, it is also possible to follow the approach of a safeguard power analysis (Perugini et al., 2014) and choose a smaller effect size to avoid underestimating the true sample size or to use the smallest effect size of interest. We will follow a safeguard power approach in the example and choose an expected effect size of *δ* = 0.2. Theoretically, it would also be possible to use a distribution of effect sizes as a data generating model which illustrates the uncertainty about the data generating process, but this option is not included in our app since it would necessitate the storage of additional precomputed Monte Carlo simulations which would dramatically slow down the app. The simulation code is, however, easy to adjust (see example on the OSF platform: https://osf.io/3f5qd/). Thus, if users like to conduct these new simulations, they can make use of our open source code.
  In the next two steps, we are going to customize the summary plot on the top of the app. The summary plot shows the expected (median) sample sizes per group for different symmetric decision boundaries given the selected effect size. Analyzing the summary plot at first can help balance evidence strength and sample size in the choice of decision boundaries.
3. Choosing an analysis prior: The summary plot allows us to check easily how much the sample size estimates depend on the choice of the prior distribution. We therefore choose both the default and the informed prior distribution on effect size.
4. Selecting additional information on the dispersion of the sample size distribution: Especially for researchers with scarce resources, it may be useful to obtain boxplot-like information on upper (and lower) bounds of the distribution. The BFDA app includes the option to display the quartiles and the 5% and 90% (see Fig. 10) quantile of the distribution. However, the question we want to answer refers mainly to the expected sample size, so we do not tick these options.
5. The summary plot shows a steep increase in expected sample sizes when decision boundaries increase. Moreover, it reveals a considerable sensitivity of the method for the choice of the analysis prior, namely considerable smaller sample sizes for informed than for default priors. For the following analyses, we will choose a relatively small symmetric decision boundary of 6, classified as “moderate evidence” by Lee and Wagenmakers (2014), assuming that it represents a reasonable starting point for a good trade-off between efficiency and informativeness. In practice, this trade-off is dependent on the available resources, on the stage of the research process, and on the desired strength of evidence.
6. Choosing an analysis prior: As before, we will choose both the default and the informed prior distribution to be able to compare the results.
7. Select information that should be displayed: We select both numeric (medians, 5%, 25%, 75%, and 95% quantiles) and pictorial representations (violin plots) of the distribution of sample sizes from the list. We could have chosen more options, but these suffice to demonstrate the case.
  The results of the Monte Carlo simulations are displayed on the right of Fig. 10. First, statistics of the distribution of sample sizes are displayed for $\mathcal {H}_{0}$ and $\mathcal {H}_{1}$. We can see that the expected sample sizes are a little smaller when the null hypothesis is correct than when the alternative hypothesis is correct. Moreover, as in the summary plot, we can see that under the alternative hypothesis, the expected sample size is smaller when the informed analysis prior is used. Remember, however, that these gains in efficiency come at the cost of higher type I error rates. Under the null hypothesis, the choice of the analysis prior has little effect on the expected sample sizes. For default priors, we can see from the quartiles tables that the 80% quantile of the sample size distribution under $\mathcal {H}_{1}$ is 235 per group. For informed priors it is 130. If planning for resources requires a definite maximum sample size (e.g., in grant applications), these are good estimates that can be used for these purposes. Due to the skewness of the distribution, our original question on the expected sample size can be answered best with the medians: For informed prior distributions, the expected sample size is 56 observations per group, for default prior distributions 76 observations (if $\mathcal {H}_{1}$ is true). The figure at the bottom of the page gives a visual representation of the distribution of sample sizes. It combines traditional violin plots with boxplots and a jitter representation of the raw data. Note that due to the extreme skewness of the distribution the y-axis is log-scaled for enhanced readability.
8. Download report: As in the fixed-N design, it is also possible to download a time-stamped dynamic report of the results. This can be achieved by clicking on the download button on the left sidebar panel. The report for the analyses of our second application example can be downloaded from https://osf.io/3f5qd/.

## Conclusions

In this article, we demonstrated the effects of the choice of priors on the results of a Bayes Factor Design Analysis (BFDA) and introduced a Shiny app which facilitates conducting a BFDA for the practical user. We provided a detailed, tutorial-style overview on the principles of BFDA and on the questions that can be answered through a BFDA and illustrated how these questions can be answered using the BFDA app.

When comparing informativeness and efficiency of designs with different analysis priors, it is clear that for most effect sizes within the typical range of psychology, fewer participants are required in the informed-prior design. This becomes especially clear in sequential designs where frequently fewer than half as many participants are required in informed-prior designs than in default-prior designs. Additionally, informed-prior designs also yield higher expected Bayes factors in a fixed-N design. This indicates that informed-prior designs are more efficient in terms of sample size and more informative in terms of expected strength of evidence than default-prior designs. However, informed-prior designs with a highest density region at small effect sizes also coincide with higher false-positive error rates compared to default-prior designs. This has to be taken into consideration when judging the informativeness of these designs.

Although comparing designs with default and informed prior distributions is sensible on a conceptual level, because it yields information on how “objective” and “subjective” designs behave in general, it is not possible to infer recommendations for or against specific prior distributions. In our opinion, the prior distributions should represent intuitions about effect sizes under the investigated hypotheses in a specific case, and not be chosen merely because of their expected effects in a design analysis. What we can infer from our results is that it pays to include available information in the prior distribution, because this enhances informativeness. However, if the true effect size differs greatly from the location of an informed prior distribution, the relative benefit of informed priors becomes negligible or can even turn into a disadvantage. It may therefore be prudent to plan such that the results will likely be compelling regardless of the prior distribution that is used.

In the paper and the accompanying app, we demonstrate the effects of the choice of analysis priors using only two prior distributions as an example. However, these results can be generalized to other default and informed analysis priors. The more the alternative hypothesis differs from the null hypothesis, the easier will it generally be to gain evidence for one or the other. This means that analysis priors which incorporate more information will generally have an efficiency advantage over relatively vague analysis priors. The specific BFDA results for other priors than the ones used in this paper can be obtained by adjusting the parameters of the analysis prior in the code of the simulation procedure which we provide online together with this paper on https://osf.io/3f5qd/ or with the *BFDA* R package starting from version 0.4.0 (Schönbrodt & Stefan, 2018).

Although BFDA is only applied to *t*-tests in this paper, the procedure of BFDA can also be generalized to other hypothesis tests. For example, similar analyses may be developed for ANOVAs (for an application, see Field et al., 2018) or for the comparison of two proportions as is popular in medicine. The main challenge here is to develop suitable data generating processes for the simulation algorithm which can be used as a design prior in the BFDA.

The BFDA approach we present in this paper shares many similarities with the generalized Bayesian power analysis approach presented by Kruschke (2013) and Kruschke and Liddell (2018) who also present a simulation-based method for design analyses in a Bayesian context. However, these authors focus on parameter estimation. Thus, instead of focusing on the Bayes factor as a measure of evidence strength, their analysis results are centered around indicators of the posterior distribution. They also propose a different standard for the definition of design priors. Specifically, they do not support the idea of a smallest effect size as a basis for the definition of design priors and use only distributed design priors. Most importantly, the current approach presented in this paper extends previous expositions of generalized Bayesian power analysis to sequential Bayesian designs.

The process of BFDA presented in this paper follows exactly the plan outlined by Schönbrodt and Wagenmakers (2018). By providing a method to plan for efficiency and informativeness in sequential designs, their approach allows for increased flexibility in research designs compared to designs based on frequentist power analyses. From a Bayesian perspective, research designs could, however, be even more flexible. Theoretically, it would be possible to ask at any point in the sampling procedure: Is the expected gain in evidence worth the effort of collecting the next datum? However, this approach requires knowledge about the expected change in Bayes factors given the collected data, about the social and financial costs of data collection, and about the utility of changes in the Bayes factor. Determining these parameters is difficult at the moment and awaits future research.

In sum, the BFDA is a powerful tool that researchers can use to balance efficiency and informativeness in the planning stage of their experiments. Our interactive Shiny app supports this endeavor by making computationally intensive Monte Carlo simulations redundant for one class of standard designs and by providing a graphical user interface, so that no programming experience is required to conduct the analyses. Although it currently covers only the independent sample *t*-test and only two prior distributions, the app can be extended to other designs, as both simulation results and source code of the app are openly accessible. With this article, we hope to have provided an accessible introduction to BFDA and have encouraged more researchers to adopt BFDA as an additional tool for planning informative experiments.

## Footnotes

1 Platt (1964) referred to the method of planning for informative results as “strong inference”. One of the main components of strong inference is “Devising a crucial experiment [...], with alternative possible outcomes, each of which will, as nearly as possible, exclude one or more of the hypotheses.” (p. 347)

2 In addition, when already large sample sizes are further increased, the incremental effect on statistical power is only small (Lachin, 1981).

3 A comprehensive introduction to Bayesian inference is beyond the scope of this article. The interested reader is referred to Etz et al., (2018) for an overview of introductory materials on Bayesian statistics.

4 For a discussion on whether optional stopping creates bias, see Schönbrodt et al., (2017, pp. 330-332) and Schönbrodt & Wagenmakers (2018, pp. 139f).

5 In frequentist statistics, there also exist tests that allow for this three-way distinction, e.g., the Wald test (Wald, 1943), but these are seldomly used in practice.

6 This property can be described by a Conditional Accuracy Function (Luce, 1986)
